# Voyages in Vacation.—XIX

**Published:** 1889-02-16

**Authors:** 


### VOYAGES IN VACATION.—XIX.

*(Continued from page 304.)*

### MORE ABOUT NORWAY.

VOSSEVANGEN is a charmingly-primitive place, with an excellent hotel, where you get good Norwegian cookery, and where the waitresses are are all dressed in the pretty national costume. This was all we had time to see of it, as we did not get there till nine p.m., and left at half-past five next morning. The first part of the drive from Vos to Stalheim is not very interesting—for Norway, at least—for here one expects each view to be more colossal than the last; but Stalheim itself, at the head of the far-famed Naerödal, is more beautiful than any description can express—one can only hold one's breath in awe, and feel thankful to have seen such glorious sights. There is a lovely little hotel at the head of the pass, the view from the balcony of which is one of the finest in Norway. The narrow valley, with towering dark rocks 3,000 to 5,000 feet high, forming an impenetrable wall on the right, rising sheer up almost straight from the valley, their pinnacles sharply cut against the brilliant blue sky; and Jordal's Knut, a conical bare mountain, 4,500 feet in height, standing out clear and grey in the sunshine in the centre of the valley, which is closed in by shadowy hills in the background, relieved here and there by streaks of white, which, as you get nearer, you find are waterfalls. This, seen as I saw it, in the morning sunlight, forms another indelible picture, beautiful as anything on earth can be, though not perhaps as impressive in its grandeur as the dark Mauranger Fiord. We descended a precipitous serpentine road, flanked by two splendid waterfalls, to the valley beneath, and drove down the narrow road to Gudvangen, about a mile above which place we were received by those of our friends who had come round by the boat and up the Naerö Fiord to fetch us. We were glad to meet them, for these great bare mountains are rather awe-inspiring even in the sunshine, and we appreciated the more the bright faces and cheery words of those who had come to welcome us—one feels so small beside the giants, and so perishable face to face with this seemingly immutable granite. It is rather a comfort to remember that "all things are not what they seem," and that even these mountains do change, although they do it with infinite leisure.

We got into our launch at Gudvangen and went down the beautiful Naerö Fiord to our beloved *Victoria*, which was waiting for us in the Sognë Fiord, about two hours' journey below. The whole way down new beauties crowded on us at every turn, and we thoroughly enjoyed our two hours' steam. After lunch the *Victoria* weighed anchor, and we proceeded down the fiord and up the Lyster and Faerlands Fiords, both branches of the Sognë, in one of which was the lovely Feigums-fos—a delicate veil of glistening water, falling in detached festoons and trailing drops, like shooting stars, or the shower of falling sparks from an exploded rocket. Just as we arrived opposite to it a gorgeous rainbow appeared, which gradually crept up the spray and produced the most exquisite transformation scene one could imagine. Nature put on her prettiest colours for our benefit, as though she liked us to admire her. At the end of, I think, the same fiord was a good view of an arm of the Justedalsbrae, the largest glacier in Europe, which comes down to within a quarter of a mile of the water at this point. I was just wishing I had not exhausted all the adjectives in my vocabulary in my various exclamations at the different views we passed, when a new one for a glacier was unexpectedly supplied by a gentleman standing near me. "I don't care for them a bit," he said, "they are such stolid brutes, there they stand and they won't budge."

We stopped a night at Balholmen, a very beautiful anchorage—still more lovely in the morning light. At length we reached the Moldë Fiord, with its lofty ring of snow-touched mountains, called at the picturesque little town for our letters, and then went over to Naes and anchored for the night. I ordered a carriage to go to the Romsdal Valley next day from a man who came on board, and early next morning as I was stepping on shore an envelope with my name upon it was shown to me, and a most polite man, who evidently imagined he was speaking excellent English, inquired: "Do any of you fellows belong to this?" We had a good carriage and two sturdy little horses for our long drive up the Romsdal Valley, which was very grand in the early light. The Rauma is a mountain torrent, fed by streams which always become waterfalls at some time during their course, and rushing over huge moss-clad boulders. The vegetation everywhere is luxuriant, beech and oak fern abounding in sheltered nooks, and forming little fairy dells of exquisite greenery among the fallen rocks by the wayside. We drove on through "heart-satisfying" beauties, with the ungainly rhinoceros horn of the Romsdal-horn on the one side, and the graceful heights of the Troll-tinder—"witches pinnacles"—on the other, and at length reached Ormeim, where we descended to see the Vermedalsfos to greater advantage. We strolled down through fjelds and mossy boulders, enriched with tufts of the feathery white saxifrage—which the Norwegians call Berg-köne—king of the mountains—until we were almost beneath the rushing three-fold torrent of the great fos. After taking our fill of this beauty, we went on to see the Slettafos, which was really the best waterfall we had ever seen. As we Victorians generally went to each place at the same time, we were continually meeting and comparing notes, and we generally decided that each thing we saw was better than the last, until we reached the Slettafos, which we thought could not be surpassed. At this point the Rauma foams and boils in an irresistible cataract over gigantic masses of fallen rock with a deafening roar, and a force that makes the solid rock tremble under one's feet. We drove back through the evening light, in which the scenery looked still more beautiful than in the morning, and arrived at our vessel about seven p.m., after a day of most thorough enjoyment.

The next day I took a drive in a carriole, and found it very comfortable, though we took an unfrequented road in the Isterdal, and drove along it at peril to life and limb. We should have been thrown out of any other vehicle, but these carrioles performed a series of leaps and bounds over the boulders in a manner truly surprising to a foreigner. We pertinaciously followed the road until we absolutely could go no further, for it disappeared altogether and left us in a bog. We went into a cottage and asked for milk. It was poor and meagrely furnished, and the people look ill-fed and very poor. They have no idea of comfort, and their life must be a hard one; but they have pleasant, kindly faces, and very intelligent eyes, and are quick to take an idea even when they do not understand English. I have forgotten to mention the Geiranger Fiord in its right place, but cannot leave it out, as it is one of the grandest of all I visited, with its bare precipitous rocks rising to a tremendous height, and the lace-like waterfalls—notable among which is the "Seven Sisters"—falling like gleaming translucent veils over the hard faces of the uncompromising cliffs. They are very ghostly in the twilight, and take all sorts of fantastic shapes, and as we turned away and left them we could almost imagine that it was some white-robed female figure we were deserting, and half expected to hear a wail of agony as she disappeared from sight!

We left for Moldë on Thursday morning, walked about the quaint little town in the early part of the day, and climbed to the Varde, a hill with a signpost, behind the town in the afternoon. The path led over boulders, and among heather, and flowers, and feathery grasses, dotted with pine trees, and carpeted with softest moss. The light on the fir trees was exquisite; and as the shadows lengthened from the westward, and the sun gradually sank towards his rest, the purple shadows grew upon the nearer mountains, and the evening glory glowed upon the everlasting hills beyond the peaceful fiord. We walked slowly and lingeringly down, and wended our way back to the ship, loth to leave the enchanted ground, but happy in having had such a perfect afternoon for our last on that beautiful shore. Our voyage home was not quite as prosperous as the outward one had been, for we encountered a storm, which somewhat spoiled the pleasure of all but the very "fittest." I thoroughly enjoyed the great green waves, and stood on the hurricane deck, clad in a sailor's oilskin, and braving the frequent drenchings for the sake of the glorious sea. Towards evening, as we neared land, the sea became calmer, and the sufferers began to revive. By next morning, when we reached Tilbury, all troubles were forgotten, and we parted company with mutual regret, congratulating each other all round on the thorough success of our expedition.

